# Methodology Aspects of Colony Maintain for a Murine Model of Amyotrophic Lateral Sclerosis (ALS) TDP-43 Proteinopathy

**DOI:** 10.3390/ani10122329

**Published:** 2020-12-07

**Authors:** César Álvaro-Alonso, Águeda Ferrer-Donato, Elizabeth Fernández-Torres, Mónica Carballo-Villa, Carmen M. Fernandez-Martos

**Affiliations:** 1 Hospital Nacional de Parapléjicos, Finca La Peraleda s/n, 45007 Toledo, Spain; calvaroa@sescam.jccm.es (C.Á.-A.); aferrerd@externas.sescam.jccm.es (Á.F.-D.); eltorres@sescam.jccm.es (E.F.-T.); mcarballov@sescam.jccm.es (M.C.-V.); 2 Wicking Dementia Research and Education Centre, College of Health and Medicine, University of Tasmania, Private Bag 143, Hobart, Tasmania 7001, Australia

**Keywords:** genetically engineered mouse (GEMs), TAR DNA binding protein (TDP-43), amyotrophic lateral sclerosis (ALS)

## Abstract

**Simple Summary:**

Rodent models of central nervous system (CNS) disorders are widely used to explore pathology and molecular mechanisms of disease. These models are a valuable tool for understanding the genetic basis of amyotrophic lateral sclerosis (ALS) research, a CNS disease that affects motor neurons in the brain and spinal cord, causing loss of voluntary muscle control. There are two different types of ALS: Sporadic (sALS), accounts for 90 to 95 percent of all cases, which means the disease seems to occur at random with no clearly associated risk factors and no family history of the disease, and familial (fALS), which is inherited and accounts for 5 to 10 percent of all cases. One example of a gene mutation in fALS is the A315T mutation to the gene encoding TDP-43. The congenic Prp-TDP43^A315T^ Tg model of ALS disease expresses full-length human *TARDBP* containing this mutation. The effects of the highly aggressive phenotype of this Tg model of human ALS, and its premature sudden death prior to full development of neurodegenerative symptoms, makes it essential to determine their reproductive pattern in order to guarantee proper colony maintenance, which is the main goal of this study.

**Abstract:**

The use of genetically engineered mouse (GEMs) models provides an unprecedented opportunity to study the genetic basis of diseases and gene function, therefore it is paramount to determine reproductive parameters that guarantee proper colony maintenance. We studied the reproductive parameters of mice hemizygous for TDP-43^A315T^ transgene, which are viable, fertile, and express a mutant human TAR DNA binding protein (hTDP-43) cDNA harboring an amino acid substitution associated with familial amyotrophic lateral sclerosis (fALS). TDP43^A315T^ mice were backcrossed to a C57Bl6/J pure background for four consecutive generations. The Tg offspring genotype were then confirmed by PCR assays. Our statistical analysis indicated there were no differences in the sex and number of pups per offspring when hemizygous female and male TDP43^A315T^ mice were backcrossed to C57Bl6/J mice. Interestingly, our results showed significant differences in the number of offspring expressing the transgene when hemizygous TDP43^A315T^ male mice were used as breeders. Therefore, our findings suggest that male TDP43^A315T^ mice transfer the transgene with a greater genetic strengths. Such is an important breeding consideration to ensure the principle of reduction in animal experimentation considering most basic research with models focuses on males and excludes female mice.

## 1. Introduction

Genetically engineered mouse (GEMs) models are essential to understand the fundamental mechanisms underlying the onset of malignance, and to discover improved methods to prevent, diagnose, and treat a wide spectrum of diseases [1]. More specifically, GEMs models are valuable tools for the genetic understanding of amyotrophic lateral sclerosis (ALS) research [2]. ALS is a motor neuron disease (MND) characterized by the selective and progressive loss of upper and lower motor neurons of the cerebral cortex, brainstem, and the spinal cord [3]. The majority of patients suffer from sporadic ALS (sALS; >90%), while only a small subset of patients suffers from inherited ALS (fALS; <10%) 20% of cases have been linked to mutations in the gene encoding copper-zinc superoxide dismutase (*SOD1*) [4], which represent the 2% of all cases of fALS. In 1993, Rosen and collaborators discover SOD1 mutations [5], which then led to the development of numerous models of Tg SOD-1 mice that simulate an ALS-like disease. However, the recent discovery of other highly relevant genetic disease-mutations such as *TAR DNA binding protein* (TDP-43) [6,7], bring new powerful GEMs models to the researchers in the field to explore the role this gene mutation plays on this complex neurological disorder.

Prp-TDP43^A315T^ Tg model of ALS disease containing the A315T mutation is seen in fALS patients [8], under the control of the mouse prion protein (PrP) promoter [9,10]. In terms of ALS pathology, these mice develop neuropathology and behavioural deficits similar to ALS and frontotemporal dementia (FTD), including cytoplasmic TDP-43 inclusions, neuroinflammation, axonal pathology, and cognitive and motor impairment. However, they do not develop neuronal loss or paralysis [9]. The initial report published by Wegorzewska et al. [9] indicated that Prp-TDP43^A315T^ mice were born at normal inheritance ratios, weighed the same as non-Tg littermates, and appeared normal up to three months of age. In this study they demonstrated that Tg Prp-TDP43^A315T^ mice have an average survival of approximately 154 ± 19 days. However, hemizygous male mice for TDP-43^A315T^ transgene die almost 1 month earlier than females [9]. Indeed, the lifespan of male hemizygous for TDP-43^A315T^ transgene appeared to be reduced when these mice were crossed with C57BL6/J pure background [11]. Coughlan et al. [12] and other research groups [11,13,14,15] determined a premature sudden death prior to full development of neurodegenerative symptoms. However, in 2014 Herdewyn et al. [16] demonstrated that the range of survival of Prp-TDP43^A315T^ mice can be extended using special diets to mitigate the intestinal dysmotility phenotype and sudden death. What is more, the results also highlighted the survival differences between Tg Prp-TDP43^A315T^ mice in females and males were still present [16]. As reported, these studies indicate Tg Prp-TDP43^A315T^ female mice have a much longer breeding window than male mice hemizygous for TDP-43^A315T^ transgene.

Therefore, determining the reproductive parameters of Prp-TDP43^A315T^ mice essential to establish the colony and to guarantee its maintenance, given the effects of the highly aggressive phenotype of this Tg line. As such, in this study we describe our findings in the characterization of the reproductive parameters of Prp-TDP43^A315T^ mice during four generations. Our results described for the first time, a greater heritage of genetic strengths of TDP-43^A315T^ transgene by Tg Prp-TDP43^A315T^ male mice compared to females, even when hemizygous male mice for this GEMs strain have a significantly shorter breeding window than females. Our results may help to redefine strategies aimed at reducing the number of animals required on research.

## 2. Methods

### 2.1. Animals

Transgenic (Tg) Prp-TDP43^A315T^ mice [9] and non-Tg C57Bl6/J control mice were used in this study. Animals were group-housed under standard housing conditions with a 12 h light–dark cycle, and food and water ad libitum. Tg Prp-TDP43^A315T^ mice were fed jellified food in pellet form (D5K52 food. Rettenmaier Ibérica, Spain) to mitigate the intestinal dysmotility phenotype and sudden death [16]. Their general heath was regularly checked as well. All experimental procedures were approved by the Animal Ethics Committee of the National Hospital for Paraplegics (HNP) (Approval No 26/OH 2018) in accordance with the Spanish Guidelines for the Care and Use of Animals for Scientific Purposes.

### 2.2. Colony Amplification for Experimental Animal Generation

Mice hemizygous for TDP-43^A315T^ transgene are viable and express a mutant human TAR DNA binding protein TDP-43) cDNA harboring an N-terminal Flag tag and an A315T amino acid substitution associated with ALS [9]. This genetic modification is not expected to alter the breeding performance. Tg Prp-TDP43^A315T^ mice breeding pair were obtained from the Jackson Laboratory (Strain No. 010700, Bar Harbor, ME, USA) to establish the colony. For colony amplification, breeding was arranged between two Tg Prp-TDP43^A315T^ male mice and non-Tg females on a C57Bl6/J pure background. Then, as hemizygous female carriers live significantly longer than hemizygous males, both female and male Tg Prp-TDP43^A315T^ mice were backcrossed to C57Bl6/J for four generations for colony maintenance. All animals expressing TDP-43^A315T^ transgene were confirmed via PCR according to the distributor’s protocol.

### 2.3. Statistical Analysis

All values are expressed as the mean ± mean standard deviation (SEM). All statistical analysis was performed using GraphPad Prism (version 8.4.2; San Diego, CA, USA). The parametric two tailed student *t* test was used for the comparisons between mean groups followed by F test in order to test the homogeneity of variances.

## 3. Results

### Colony Amplification for Experimental Animal Generation of Tg Prp-TDP43^A315T^ Mice

**Figure 1:**
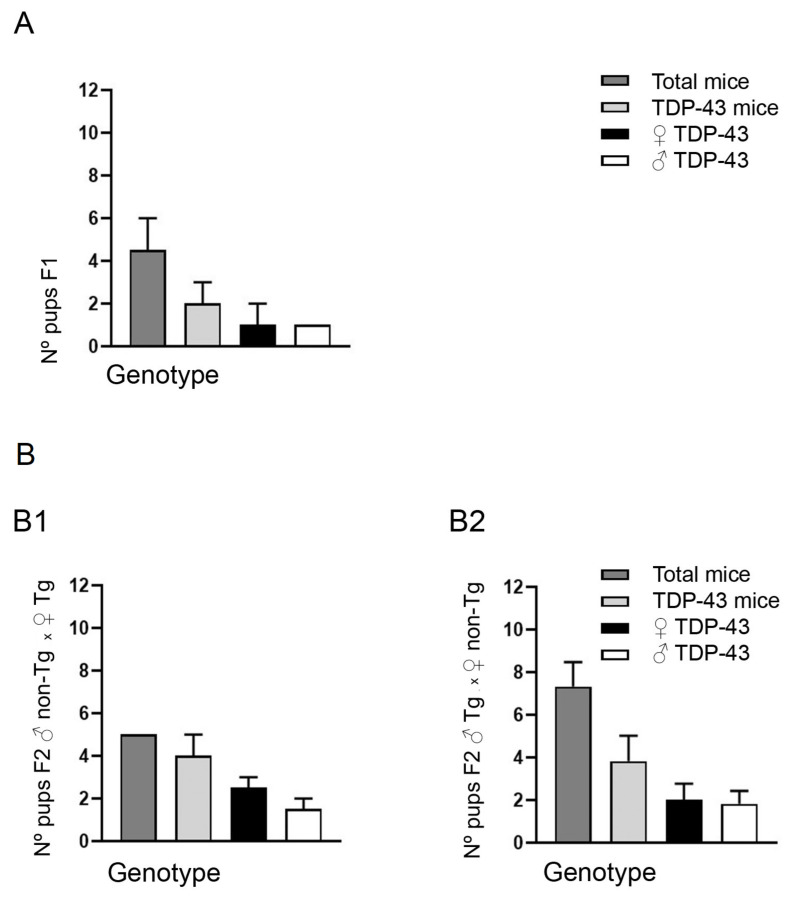
Analysis of prion protein (PrP) TAR DNA binding protein (TDP-43)^A315T^ transmission across F1 and F2 generation. (**A**) F1 generation, data obtained from 2 crosses of ♂ TDP-43^A315T^ × ♀ C57BL6/J. (**B**) F2 generation, data obtained from 2 crosses of ♂ C57BL6/J × ♀ TDP-43^A315T^(**B1**) and F2 generation, data obtained from 6 crosses of ♂ TDP-43^A315T^ × ♀ C57BL6/J (**B2**). Y axis measures pups per litter mean and X axis represents the total number of mice (Total mice), Tg or TDP-43^A315T^ mice (TDP-43 mice), Tg female or TDP-43^A315T^ female mice (♀ TDP-43) and Tg male or TDP-43^A315T^ male mice (♂ TDP-43) on represented generation. Bar graphs represent the mean ± SEM. Abbreviations: Prp, prion protein; F1, first filial generation; F2, second filial generation; Tg, transgenic; SEM, standard error of the mean.

Breeder pairs were ordered from Jackson Laboratory (Bar Harbor, ME, USA) to establish a colony at the animal facilities of the HNP (Spain). For the initial colony amplification, mating was arranged between two breeder pair Tg Prp-TDP43^A315T^ male mice (called F0) delivered by Jackson Laboratory, and two C57Bl6/J females, with the goal of maintaining the same background. Subsequently, both F1 offspring (*n* = 9 mice in total) were subjected to genotype analysis 21 days after birth. Followed by DNA isolation and PCR analysis (data not showed), our results confirmed only four mice expressed TDP-43^A315T^ transgene (2 males and 2 females), from a total of 4 females and 5 males pups tested (Figure 1A). Therefore, only 44% of the F1 progeny were hemizygous for Prp-TDP43^A315T^ transgene. Values (mean ± SEM) are shown in Table S1.

**Figure 2:**
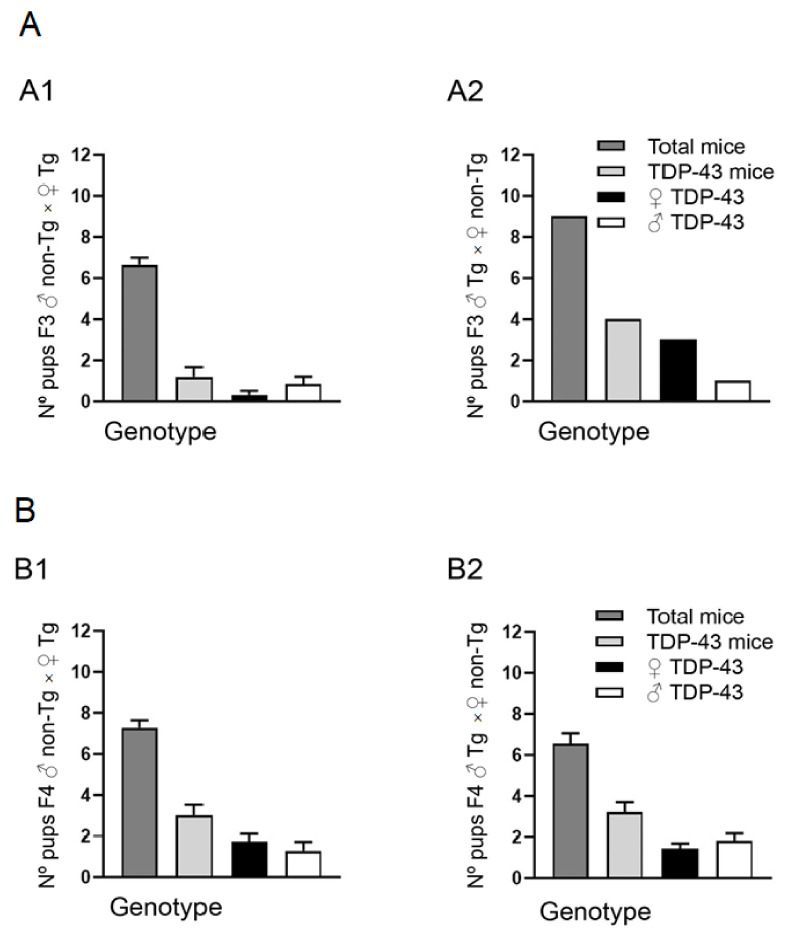
Analysis of Prp-TDP-43^A315T^ transmission across F3 and F4 generation. (**A**) F3 generation, data obtained from 15 crosses of ♂ C57BL6/J × ♀ TDP-43^A315T^ (**A1**) and F3 generation, data obtained from 1 cross of ♂ TDP-43^A315T^ × ♀ C57BL6/J (**A2**). (**B**) F4 generation, data obtained from 7 crosses of ♂ C57BL6/J × ♀ TDP-43^A315T^. (**B1**) and F4 generation, data obtained from 21 crosses of 2 ♂ TDP-43^A315T^ × 21 ♀ C57BL6/J (**B2**). Y axis measures pups per litter mean and X axis represents the total number of mice (Total mice), Tg or TDP-43^A315T^ mice (TDP-43 mice), Tg female or TDP-43^A315T^ female mice (♀ TDP-43) and Tg male or TDP-43^A315T^ male mice (♂ TDP-43) on represented generation. Bar graphs represent the mean SEM. Abbreviations: Prp, prion protein; F3, third filial generation; F4, fourth filial generation; Tg, transgenic; ±SEM, standard error of the mean.

Then, two Tg Prp-TDP43^A315T^ mice (2 male and 2 female), from F1 respectively, were backcrossed to 8 C57Bl6/J mice. From this second generation (F2) we obtained 8 litters, one per each Tg Prp-TDP43^A315T^ female mice, and three per each Tg Prp-TDP43^A315T^ male mice. Interestingly, following PCR assays, our analysis indicated that 80% of the progeny (8 positive mice/10 mice tested) were positive for the transgene when Tg Prp-TDP43^A315T^ female mice were backcrossed to C57Bl6/J male mice (Figure 1B(B1)). This is compared to the 52% of mice expressing TDP-43^A315T^ transgene when hemizygous Prp-TDP43^A315T^ male mice were used as breeders (23 positive mice/ 44 mice tested) (Figure 1B(B2)). Values (mean ± SEM) are shown in Table S2. Therefore, with these results, and considering that it is considerably more difficult to maintain the colony using Tg Prp-TDP43^A315T^ male mice, the third generation (F3) breeding was arranged between 13 Tg Prp-TDP43^A315T^ female mice and 1 C57Bl6/J male mice. Surprisingly, although we obtained 15 litters from Tg Prp-TDP43^A315T^ female mice with 100 mouse pups, following PCR assays (data not showed), our results confirmed 82 non-Tg mice and only 18 mice (13 females and 5 males) were positive for TDP-43^A315T^ transgene (Figure 2A(A1)). Additionally, one Tg Prp-TDP43^A315T^ male mice were backcrossed to one C57Bl6/J female mice. Following PCR assays of the offspring (*n* = 9 mice in total), our analysis indicated 4 positive mice for the transgene, 3 males and 1 female (Figure 2A(A2)). Notably, this result indicates that heritability of the TDP-43^A315T^ transgene were decreased from the 80% to 17% from F2 to F3 when Tg Prp-TDP43^A315T^ female mice were backcrossed with C57Bl6/J male mice, while the heritability of the TDP-43^A315T^ transgene was 44% when hemizygous males were used as breeders (Figure 2A(A2)). Values (mean ± SEM) are shown in Table S3.

To obtain the F4, 7 females and 2 males Tg Prp-TDP43^A315T^ mice, respectively, were backcrossed to 28 C57Bl6/J mice. In this F4, we obtained 28 litters with a total 189 pups. After genotype procedures, 100 mice were non-Tg mice, and only 89 mice (47 females and 42 males) were positive for TDP-43^A315T^ transgene by PCR assays. Our data indicated that the 41% of the progeny (21/ 51 pups tested) were positive for TDP-43^A315T^ transgene when Tg Prp-TDP43^A315T^ female mice were backcrossed to C57Bl6/J male mice (Figure 2B(B1)), compared to the 49% (68/ 138 pups tested) of mice expressing TDP-43^A315T^ transgene when hemizygous Prp-TDP43^A315T^ male mice were used as breeders (Figure 2B(B2)). Values (mean ± SEM) are shown in Table S4.

Therefore, in total, we obtained 24 litters when Tg Prp-TDP43^A315T^ female mice were backcrossed to C57Bl6/J male mice, with a total number of 161 offspring, and 47 Tg Prp-TDP43^A315T^ mice (30% of the progeny) y 30 litters when Tg Prp-TDP43^A315T^ male mice were backcrossed to C57Bl6/J female, with a total number of 200 offspring, and 99 Tg Prp-TDP43^A315T^ mice (50% of the progeny) (Table 1). The mean number pups per litter was similarly independent of the sex of the Tg Prp-TDP43^A315T^ mice used. However, our data indicated a greater heritage genetic strengths of TDP-43^A315T^ transgene by Tg Prp-TDP43^A315T^ male mice compared to female.

## 4. Discussion

In recent years, there have been great advances in the use of genetically modified mice to study pathophysiological mechanisms involved in ALS [2]. GEMs have been the predominant experimental tools, and have allowed rapid progress in our understanding of the role of several proposed gene mechanisms in ALS pathogenesis [17]. In general, when working GEMs lines that remain in heterozygosity, it is important to know reproductive parameters in order to design reproduction strategies that guarantee the maximum possible number of animals carrying the desired transgene [18]. However, investigators often avoid properly evaluating this aspect in the favor their usefulness for understanding gene function and genetic basis of diseases. Consequently, very few studies analyze the reproductive parameters of GEMs line. To our knowledge, thus far no study has previously evaluated this aspect in Prp-TDP43^A315T^ murine models of FTLD/ALS TDP-43 proteinopathy. In this context, we analyzed the reproductive parameters of mice hemizygous for TDP-43^A315T^ transgene, which were viable and fertile. Our results confirmed a greater heritage of genetic strengths of TDP-43^A315T^ transgene by Tg Prp-TDP43^A315T^ male mice compared to females, which may help to redefine strategies aimed at reducing the number of animals required on ALS research.

In our study, both female and male Tg Prp-TDP43^A315T^ mice were backcrossed to C57Bl6/J pure background during four consecutive generations. The Tg offspring genotype were confirmed by PCR assays. In total, we analyzed 54 litters, with a variable number of pups per each birth, 30 of them obtained when 7 Tg Prp-TDP43^A315T^ male mice were backcrossed to C57Bl6/J female, and 24 when 21 Tg Prp-TDP43^A315T^ female mice were backcrossed to C57Bl6/J male, respectively. A total of 361 pups were weaned between 21 and 26 days of age. In all cases, we collected the samples for genotyping at the time of weaning. Following PCR assays, our results confirmed important differences in the number of positive mice for TDP-43^A315T^ transgene, depending on the sex of the progenitor used to backcrossing with non-Tg C57Bl6/J controls. Indeed, the 49.50% of mice expressing TDP-43^A315T^ transgene were positive when Tg Prp-TDP43^A315T^ male mice were backcrossed to C57Bl6/J female, compared to the 19% when Tg Prp-TDP43^A315T^ female mice were used. Therefore, our study suggests a greater heritage genetic strengths of TDP-43^A315T^ transgene by Tg Prp-TDP43^A315T^ male mice compared to females, even when hemizygous male mice for this GEMs strain have a significantly shorter breeding window. In this context, our study may help to redefine strategies for the experimental use of Tg Prp-TDP43^A315T^ mice with the aim to reduce the number of animals required on research. This is important as in basic research with animal models the focus has been on males and excluded females [19]. In the field of our study, and in general in neuroscience, when researchers use GEMs mice, males are preferred in order to avoid the effects of the estrous cycle on the experimental results [20,21,22,23]. Indeed, the sex of animal subjects is omitted in 22–42% of articles in neuroscience, and results of studies in males are often generalized to females without justification. This presents a problem as, in many cases, including our study, sex has proven an important variable to take in consideration.

In summary, our study is the first to describe importance of reproductive parameters of Tg Prp-TDP43^A315T^ mice. Our findings indicate that investigators need to redefine strategies for the experimental use of Tg Prp-TDP43^A315T^ mice as gender differences is an important aspect of breeding to take into consideration to ensure the principle of reduction in animal experimentation. In addition, our data will also significantly aid researchers to ensure and reduce the general maintenance cost of the colony, including animal facility laboring costs, less sampling for genotyping and, subsequently, fewer negative animals will be slaughtered. Nonetheless, future studies will be necessary to determine not only the genetic mechanism involved in the heritability for TDP-43^A315T^ transgene, but also to further study the contribution of the nutrition as a key factor for the genetic inheritance on this murine model of FTLD/ALS TDP-43 proteinopathy.

## Figures and Tables

**Table 1:** Analysis of Prp TDP-43^A315T^ transmission across F1–F4 generation.

Genotype	♂ TDP-43^A315T^ × ♀ C57Bl6/J		♂ C57Bl6/J × ♀ TDP-43^A315T^		Student’s *t* Sig.
	*M*	*SEM*	*M*	*SEM*	
Size litter	6.67	0.44	6.71	0.26	0.939
TDP-43^A315T^ mice	3.30	0.40	1.96	0.39	0.022 *
TDP-43^A315T^ male	1.57	0.24	0.92	0.23	0.063
TDP-43^A315T^ female	1.73	0.29	1.04	0.24	0.084

This table indicates the data recollected during 4 generations (F1–F4) of Prp TDP-43^A315T^ The first column represents mean and SEM of the offspring obtained from ♂ TDP-43^A315T^ × ♀ C57Bl6/J crosses (*n* = 30 litters, 200 offspring). The second column represents data obtained from ♂ C57Bl6/J × ♀ TDP-43^A315T^ crosses (*n* = 24 litters, 161 offspring). The last column represents the signification for the student’s *t* test. Student´s *t* test was used for the comparisons between ♂ TDP-43^A315T^ × ♀ C57Bl6/J group and ♂ C57Bl6/J × ♀ TDP-43^A315T^ group (* *p* < 0.05, *t* test). Abbreviations: Prp, prion protein; F1, first filial generation; F4, fourth filial generation; Tg, transgenic; SEM, standard error of the mean; M, mean.

## Supplementary Materials

The following are available online at https://www.mdpi.com/2076-2615/10/12/2329/s1, Table S1: Analysis of Prp TDP-43^A315T^ transmission in F1 generation, Table S2: Analysis of Prp TDP-43A315T transmission in F2 generation, Table S3: Analysis of Prp TDP-43A315T transmission in F3 generation, Table S4: Analysis of Prp TDP-43^A315T^ transmission across F1–F4 generation.
