# Supplementary material for: Methodology Aspects of Colony Maintain for a Murine Model of Amyotrophic Lateral Sclerosis (ALS) TDP-43 Proteinopathy
Source: Animals (Basel). 2020 Dec 7;10(12):2329. doi: 10.3390/ani10122329 (PMC7762410; doi:10.3390/ani10122329)
Supplement: Supplementary file 1 [file animals-10-02329-s001.pdf]

animals

Table S1. Analysis of Prp TDP-43^{A315T} transmission in F1 generation.

Genotype	♂ TDP-43^{A315T} × ♀ C57Bl6/J	
	M	*SEM*
Size litter	4.50	1.50
TDP-43^{A315T} mice	2.00	1.00
TDP-43^{A315T} male	1.00	1.00
TDP-43^{A315T} female	1.00	0.00

Table S2. Analysis of Prp TDP-43^{A315T} transmission in F2 generation.

Genotype	♂ TDP-43^{A315T} × ♀ C57Bl6/J		♂ C57Bl6/J × ♀ TDP-43^{A315T}	
	M	*SEM*	*M*	*SEM*
Size litter	7.33	1.15	5.00	0.00
TDP-43^{A315T} mice	3.83	1.19	4.00	1.00
TDP-43^{A315T} male	2.00	0.78	2.50	0.50
TDP-43^{A315T} female	1.83	0.60	1.50	0.50

Table S3. Analysis of Prp TDP-43^{A315T} transmission in F3 generation.

Genotype	♂ TDP-43^{A315T} × ♀ C57Bl6/J		♂ C57Bl6/J × ♀ TDP-43^{A315T}	
	M	*SEM*	*M*	*SEM*
Size litter	9.00		6.67	0.33
TDP-43^{A315T} mice	4.00		1.2	0.47
TDP-43^{A315T} male	3.00		0.33	0.19
TDP-43^{A315T} female	1.00		0.87	0.33

Table S4. Analysis of Prp TDP-43^{A315T} transmission across F1–F4 generation.

Genotype	♂ TDP-43^{A315T} × ♀ C57Bl6/J		♂ C57Bl6/J × ♀ TDP-43^{A315T}	
	M	*SEM*	*M*	*SEM*
Size litter	6.57	0.50	7.29	0.36
TDP-43^{A315T} mice	3.24	0.46	3.00	0.53
TDP-43^{A315T} male	1.43	0.25	1.71	0.42
TDP-43^{A315T} female	1.81	0.38	1.29	0.42

Publisher's Note: MDPI stays neutral with regard to jurisdictional claims in published maps and institutional affiliations.

© 2020 by the authors. Licensee MDPI, Basel, Switzerland. This article is an open access article distributed under the terms and conditions of the Creative Commons Attribution (CC BY) license (http://creativecommons.org/licenses/by/4.0/).
